# Zearalenone Induces MLKL-Dependent Necroptosis in Goat Endometrial Stromal Cells via the Calcium Overload/ROS Pathway

**DOI:** 10.3390/ijms231710170

**Published:** 2022-09-05

**Authors:** Yanyan Yi, Kangkang Gao, Liang Zhang, Pengfei Lin, Aihua Wang, Yaping Jin

**Affiliations:** 1 College of Veterinary Medicine, Northwest A&F University, Xianyang 712100, China; 2 Key Laboratory of Animal Biotechnology of the Ministry of Agriculture, Northwest A&F University, Xianyang 712100, China

**Keywords:** zearalenone (ZEA), goat endometrial stromal cells (gESCs), necroptosis, Ca^2+^ overload, reactive oxygen species (ROS), mitochondria damage

## Abstract

Zearalenone (ZEA) is a fungal mycotoxin known to exert strong reproductive toxicity in animals. As a newly identified type of programmed cell death, necroptosis is regulated by receptor-interacting protein kinase 1 (RIPK1), receptor-interacting protein kinase 3 (RIPK3), and mixed-lineage kinase domain-like pseudokinase (MLKL). However, the role and mechanism of necroptosis in ZEA toxicity remain unclear. In this study, we confirmed the involvement of necroptosis in ZEA-induced cell death in goat endometrial stromal cells (gESCs). The release of lactate dehydrogenase (LDH) and the production of PI-positive cells markedly increased. At the same time, the expression of RIPK1 and RIPK3 mRNAs and P-RIPK3 and P-MLKL proteins were significantly upregulated in ZEA-treated gESCs. Importantly, the MLKL inhibitor necrosulfonamide (NSA) dramatically attenuated gESCs necroptosis and powerfully blocked ZEA-induced reactive oxygen species (ROS) generation and mitochondrial dysfunction. The reactive oxygen species (ROS) scavengers and N-acetylcysteine (NAC) inhibited ZEA-induced cell death. In addition, the inhibition of MLKL alleviated the intracellular Ca^2+^ overload caused by ZEA. The calcium chelator BAPTA-AM markedly suppressed ROS production and mitochondrial damage, thus inhibiting ZEA-induced necroptosis. Therefore, our results revealed the mechanism by which ZEA triggers gESCs necroptosis, which may provide a new therapeutic strategy for ZEA poisoning.

## 1. Introduction

Zearalenone (ZEA), a secondary metabolite produced by the fungi of *Fusarium genera*, widely contaminates cereals and foodstuffs, including corn, barley, oats, and wheat [1]. Due to the stability of its structure, ZEA degrades slowly during storage and is difficult to break down by grinding, high-temperature treatments or wet milling of grain, resulting in the frequent incidence of mycotoxicosis in livestock and poultry farming. ZEA targets multiple organs, causing genotoxicity, hepatotoxicity, immunotoxicity, and reproductive toxicity, which result in substantial economic losses [2]. Among these effects, various changes and disorders related to the reproductive system caused by ZEA are particularly important [3]. With the estrogen-like chemical structure, ZEA can affect growth, estrus cycle, and pregnancy by competitively binding to the estrogen receptor [4]. Recent studies revealed that ZEA and its metabolites can disrupt pregnancy events, including fertilization rate, preimplantation embryo development and transportation, embryo implantation, and potential placental development, ultimately affecting pregnancy outcomes [5,6]. Kunishige and Zhao reported that ZEA blocked pregnancy establishment and maintenance, resulting in delayed embryo implantation in early pregnancy mice [7,8]. In studies of female rats, Gao et al. showed that gestational exposure to 20 mg/kg ZEA disrupted hormone secretions in parent and offspring rats and decreased the survival rate of the offspring [9]. Similarly, Zhao et al. demonstrated that ZEA affected pregnant rats and the development of fetuses and impaired the reproductive function of offspring rats [10]. Due to its harm to livestock and poultry, ZEA has been identified as a potential threat to public safety, highlighting the urgent need to elucidate the toxicological mechanism of ZEA.

As the site of embryonic attachment and growth, the endometrium is strongly associated with placental formation, nutrient transport, and immune regulation. The endometrium consists mainly of two cells: endometrial epithelial cells and endometrial stromal cells. Endometrial stromal cells play a critical role in endometrial immunity by regulating the expression of immune-related proteins in epithelial cells. In addition, endometrial stromal cells mediate embryo implantation and uterine involution through their proliferative and differentiation capacity. Hence, the functional and structural integrities of the endometrium are critical for a successful pregnancy. Wu et al. revealed that exposure to ZEA during embryo implantation could reduce embryo size and increase dysontogenesis rates by inducing endometrial cell apoptosis in the gilts [11]. However, the other mechanism underlying the effects of ZEA on the endometrium remains to be fully elucidated.

Recent studies have clearly revealed multiple molecular mechanisms of ZEA-induced cell damage, mainly including its effects on cell proliferation and differentiation, the cell cycle, DNA damage, and apoptosis [12]. In addition, autophagy, endoplasmic reticulum stress and oxidative stress have also been shown to be involved in the toxicological mechanisms of ZEA [13,14]. Necroptosis is a mode of programmed cell death and is mediated via receptor-interacting protein kinase 1 (RIPK1), RIPK3, and mixed lineage kinase region-like kinases (MLKL) [15]. When caspase-8 activity is inhibited, the kinase activity of RIPK1 is activated, promoting its phosphorylation, which in turn activates RIPK3 interactions via RIP homotypic interaction motifs (RHIMs) to form the necrosome. MLKL is then further phosphorylated and multimerized by the necrosome, followed by translocation to the cell membrane and disruption of plasma membrane integrity, resulting in necroptosis [16]. Yu et al. found that ZEA induced RAW264.7 cell death mainly via necrosis rather than apoptosis [17], and Wang showed that high-dose ZEA treatments increased the proportion of PI-positive cells [18]. However, whether necroptosis is involved in ZEA-induced cell death remains an open question. In our previous study, we explored the mechanism of ZEA injury in primary mouse ovarian granulosa cells and used flow cytometry to show that ZEA causes cell necrosis. Furthermore, KEGG enrichment analysis of proteomics data revealed significant enrichments in the necroptotic pathway. Therefore, we hypothesized that necroptosis contributes to ZEA-induced cell death, although the specific mechanism remains to be elucidated.

In this study, we explored the role of necroptosis in ZEA-induced endometrial injury and clarified its molecular mechanism. These data will provide a new scientific foundation for developing strategies to alleviate the pathogenic effects of ZEA and provide a basis for targeted protective drug screening.

## 2. Results

### 2.1. ZEA Induces gESCs Necrosis

**Figure 1:**
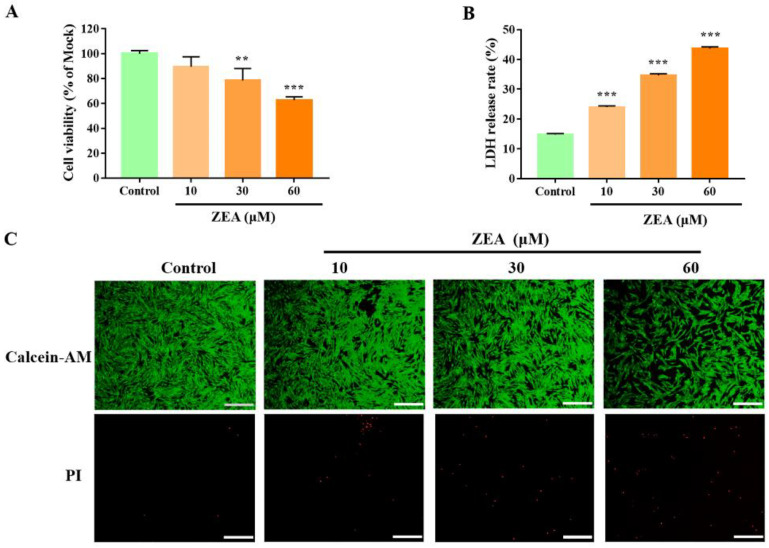
ZEA induces gESCs necrosis. Cells were treated with different concentrations (10, 30, and 60 μM) of ZEA for 24 h. (**A**) CCK-8 assays of ZEA-treated gESCs. (**B**) LDH release cytotoxicity assays of ZEA-treated gESCs. (**C**) Cell death was assessed by the Calcein-AM/PI staining. Scale bar, 100 μm. ** *p* < 0.01, *** *p* < 0.001 vs. control group.

The effect of ZEA on gESCs viability was evaluated by a CCK-8 assay. Compared to the mock group, after being treated for 24 h, ZEA significantly decreased cell viability in a dose-dependent manner (Figure 1A). Next, we investigated whether ZEA induces necroptosis. The release of LDH, an indicator of cytotoxicity, was significantly upregulated in a dose-dependent manner after ZEA treatment (Figure 1B). We confirmed our results by observing an enhanced proportion of PI-positive cells, indicating that these cells underwent necrosis (Figure 1C). These data suggested that ZEA induces the significant necrotic death of gESCs.

### 2.2. MLKL-Mediated Necroptosis Contributes to ZEA-Induced gESCs Death

**Figure 2:**
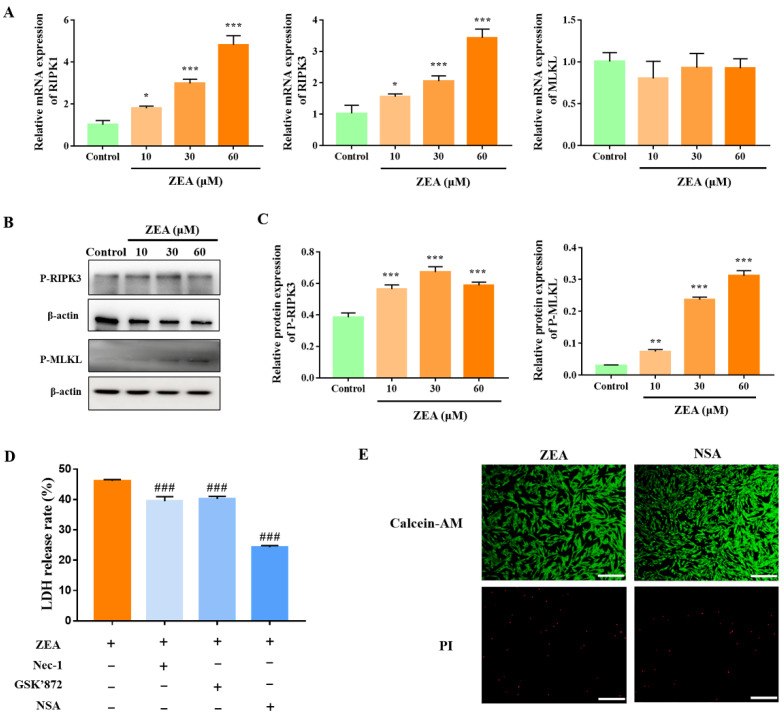
ZEA induces MLKL-mediated necroptosis in gESCs. A-B Cells were treated with different concentrations (10, 30, and 60 μM) of ZEA for 24 h. (**A**) RT-qPCR analysis of the mRNA levels of necroptosis-related proteins. (**B**) Western blot analysis of the expressions of P-RIPK3 and P-MLKL proteins. (**C**) Quantification of P-RIPK3 and P-MLKL expression, with β-actin as an internal control. (**D**) Cells were pretreated with the RIPK1 inhibitor Nec-1 (10 μM), RIPK1 inhibitor GSK’872 (5 μM), or MLKL inhibitor NSA (2.5 μM) for 2 h before treatment with 60 μM ZEA for 24 h. LDH release was evaluated using a cytotoxicity LDH assay kit. (**E**) Cells were pretreated with NSA for 2 h before treatment with ZEA for 24 h. Cell death was detected by Calcein-AM/PI staining. Scale bar, 100 μm. * *p* < 0.05, ** *p* < 0.01, *** *p* < 0.001 vs. control group. ### *p* < 0.001 vs. ZEA group.

To investigate the involvement of necroptosis in regulating ZEA-induced necrosis of gESCs, we analysed the expression of necroptosis-associated proteins by Western blotting and RT-qPCR. Compared to the mock group, ZEA significantly increased the mRNA levels of RIPK1 and RIPK3 in gESCs (Figure 2A) (*p* < 0.05). Western blot analysis demonstrated that ZEA significantly increased the phosphorylation of RIPK3 and MLKL (Figure 2B,C) (*p* < 0.05). Pre-treatment with specific inhibitors of RIPK1, RIPK3, and MLKL necrostatin-1 (Nec-1, 10 μM), GSK’872 (5 μM), and necrosulfonamide (NSA, 2.5 μM), respectively, significantly alleviated ZEA-induced LDH release, with NSA having the most marked effect (Figure 2D) (*p* < 0.001). Moreover, NSA significantly reduced the proportion of the PI-positive cells induced by ZEA treatment (Figure 2E). These results suggested that ZEA induced necroptosis in gESCs and that MLKL mainly regulated the process.

### 2.3. ZEA Induces ROS-Mediated Mitochondrial Damage in gESCs

**Figure 3:**
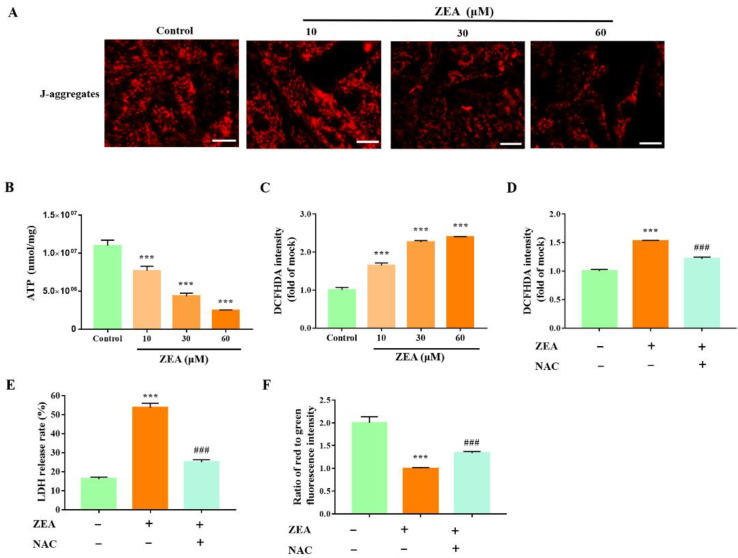
ZEA induces ROS-mediated mitochondrial dysfunction in gESCs. A-C Cells were treated with ZEA at the indicated concentrations (10, 30, and 60 μM) for 24 h. (**A**) Mitochondrial membrane potential was visualized using a probe for J-aggregates. Scale bar, 20 μm (**B**) The ATP content was detected using an ATP assay kit. (**C**) ROS production in ZEA-treated cells was evaluated by fluorescence analysis of DCFH-DA. D-G Cells were pre-incubated with the ROS scavenger NAC (50 μM) for 2 h before treatment with ZEA (60 μM) for 24 h. (**D**) The ROS generation was evaluated by fluorescence analysis of ROS-DCFH-DA. (**E**) LDH release was assessed using a cytotoxicity LDH assay kit. (**F**) Mitochondrial membrane potential was assessed by fluorescence analysis of JC-1 staining. *** *p* < 0.001 vs. control group. ### *p* < 0.001 vs. ZEA group.

Previous studies have shown that ZEA increases ROS production in various cells [14]. In the present study, we observed a significant decrease in ATP content and a significant decrease in J-aggregates in ZEA-treated cells (Figure 3A,B) (*p* < 0.001). Moreover, ZEA induced the overproduction of ROS, as demonstrated by DCFH-DA fluorescence (Figure 3C) (*p* < 0.001). Furthermore, to clarify the effect of ROS on mitochondrial damage, the cells were pretreated with the ROS scavenger acetylcysteine (NAC, 50 μM) and then treated with ZEA. NAC pretreatments significantly alleviated the ZEA-induced generation of ROS and cytotoxicity (Figure 3D, E) (*p* < 0.001) and inhibited the ability of ZEA to decrease the mitochondrial membrane’s potential (Figure 3F) (*p* < 0.001). These results demonstrated that ZEA induced significant mitochondrial damage in gESCs, and reproduction contributed to ZEA-induced mitochondrial dysfunction and cell death.

### 2.4. MLKL Contributes to the Regulation of ZEA-Induced ROS Production and Mitochondrial Dysfunction in gESCs

**Figure 4:**
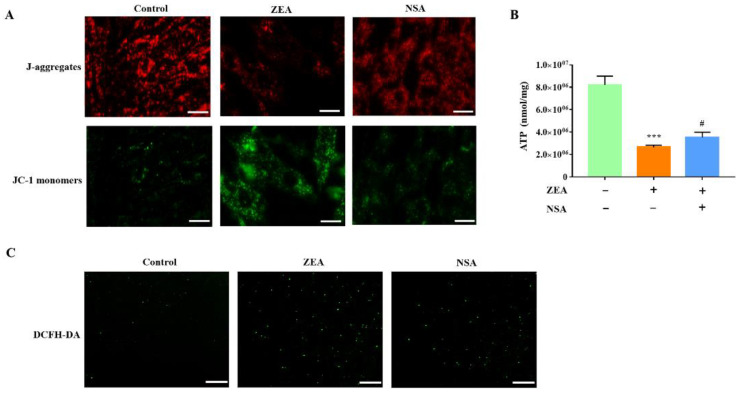
ZEA induces MLKL-mediated ROS overproduction and mitochondrial dysfunction in gESCs. Cells were pretreated with NSA (2.5 μM) for 2 h before treatment with ZEA (60 μM) for a further 24 h. (**A**) Mitochondrial membrane potential was measured by fluorescence microscope analysis of JC-1 staining. Scale bar, 20 μm. (**B**) ATP content was determined using an ATP assay kit. (**C**) ROS generation was detected by DCFH-DA staining. Scale bar, 100 μm. *** *p* < 0.001 vs. control group. # *p* < 0.05 vs. ZEA group.

Extensive studies revealed that ROS production is associated with necroptosis in various cell types [19,20]. Hence, we further investigated the relationship between gESCs necroptosis and ROS. As shown in Figure 4A, NSA pretreatment significantly reduced the ZEA-induced production of the JC-1 monomer. ATP consumption was also reduced considerably in the NSA treatment group (Figure 4B) (*p* < 0.05). Moreover, we observed weak fluorescence signals in the NSA-pretreated cells, indicating that NSA inhibited the production of ROS (Figure 4C). Overall, these results provided evidence that MLKL contributes to the ZEA-induced mitochondrial damage in gESCs by promoting ROS production.

### 2.5. MLKL Promotes ROS Overproduction by gESCs through Cytosolic Ca^2+^ Accumulation

**Figure 5:**
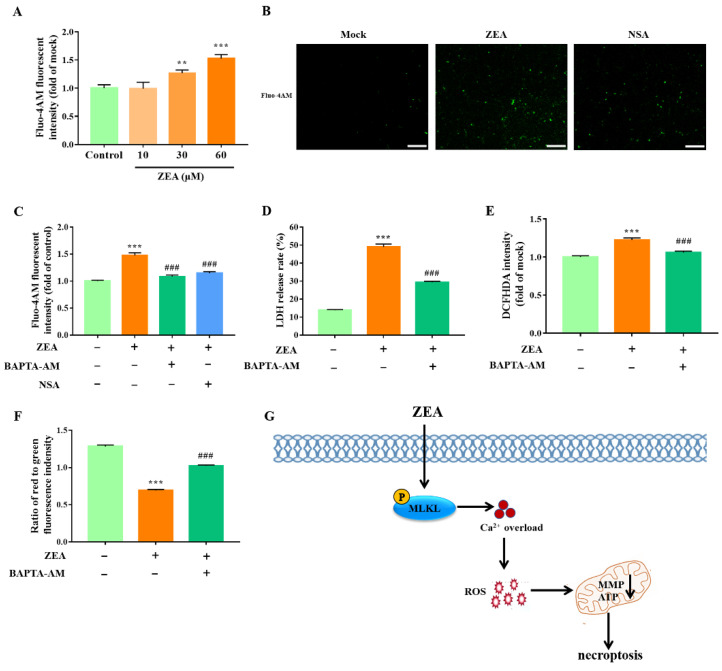
MLKL promotes ROS generation and mitochondrial dysfunction in gESCs by inducing Ca^2+^ overload. (**A**) Cells were treated with ZEA at the indicated concentrations (10, 30, and 60 μM) for 24 h before Ca^2+^ levels were assessed by Fluo-4AM staining. (**B**) Cells were pretreated with NSA (2.5 μM) before treatment with ZEA (60 μM) for 24 h. Fluo-4AM staining was performed to assess Ca^2+^ levels. Scale bar, 100 μm. (**C**) Cells were pretreated with NSA (2.5 μM) and Ca^2+^ chelation agent BAPTA-AM (1 μM) for 2 h, respectively, before treatment with ZEA (60 μM) for a further 24 h. Ca^2+^ levels were detected by Fluo-4AM staining, and a fluorescence microplate read the data. D-G Cells were pretreated with BAPTA-AM (1 μM) for 2 h before treatment with ZEA (60 μM) for a further 24 h. (**D**) LDH release was assessed using a cytotoxicity LDH assay kit. (**E**) ROS generation was measured by DCFH-DA, and the fluorescence intensity was present by a fluorescence microplate. (**F**) The mitochondrial membrane potential was assessed by JC-1 staining, and the data were quantified using a fluorescence microplate. (**G**) Schematic model delineating the pathway of the necroptosis caused by ZEA in gESCs. ** *p* < 0.01, *** *p* < 0.001 vs. control group. ### *p* < 0.001 vs. ZEA group.

Based on our results, we subsequently analyzed the molecular mechanism by which MLKL-mediated necroptosis promotes ROS production by gESCs. Reports showed that MLKL is closely related to intracellular Ca^2+^ accumulation. First, we investigated the effect of ZEA on intracellular Ca^2+^ by Fluo-4AM fluorescence staining. As shown in Figure 5A, we observed a significant increase in Fluo-4AM fluorescent intensity in ZEA-treated cells (*p* < 0.05), indicating that ZEA induced intracellular Ca^2+^ accumulation in a dose-dependent manner. Furthermore, we confirmed that pretreatment of gESCs with NSA inhibited the ZEA-induced Ca^2+^ accumulation (Figure 5B,C) (*p* < 0.001), indicating that MLKL contributes to the regulation of cytosolic Ca^2+^ accumulation, leading to overloading. Next, to demonstrate the role of Ca^2+^ in ZEA-induced necroptosis, we used the Ca^2+^ chelation agent BAPTA-AM (1 μM) to reduce cytosolic Ca^2+^ accumulation. We found that BAPTA-AM pretreatment decreased the ZEA-induced release of LDH in gESCs (Figure 5D) (*p* < 0.001), indicating that Ca^2+^ contributes to ZEA-induced necroptosis. Moreover, the analysis of DCFH-DA fluorescence intensity revealed that BAPTA-AM significantly protected cells from ROS attacks (Figure 5E) (*p* < 0.001). In addition, BAPTA-AM pretreatment inhibited the ability of ZEA to reduce the mitochondrial membrane potential in gESCs, as demonstrated by the increased ratio of red to green fluorescent intensity (Figure 5F) (*p* < 0.001). These experiments confirmed that MLKL-mediated necroptosis promotes the ZEA-induced ROS generation and mitochondrial dysfunction in gESCs caused by Ca^2+^ accumulation.

## 3. Discussion

It is well known that multiple cell death pathways, such as apoptosis and autophagy, are involved in the mechanism of ZEA toxicity [21,22]. However, whether necroptosis is a mechanism of cell death in ZEA-triggered toxicity remains unclear. In the present study, we first confirmed that ZEA caused necroptosis in gESCs via a mechanism dependent mainly on the MLKL pathway. Furthermore, our results demonstrated that ZEA-induced gESCs necroptosis contributed to mitochondrial dysfunction by promoting ROS generation and intercellular Ca^2+^ overload. These findings advance our understanding of the molecular signaling pathways underlying necroptosis induced by ZEA.

Previous studies demonstrated that ZEA induced RAW264.7 cell death mainly by necrosis, rather than apoptosis, through AIF-mediated and ROS-dependent pathways [17]. However, the ability of ZEA to induce necrosis in germ cells was not assessed. Wang et al. also reported that the flow cytometric analysis of ZEA-treated primary Leydig cells revealed an increase in the proportion of PI-positive cells [18]. However, the mechanism of necrosis was not explored further. In the current study, we revealed that ZEA induced significant gESCs cytotoxicity in a dose-dependent manner, as demonstrated by the decreased cell viability. LDH-release assays and dead/live cell staining for evaluating cell necrosis [23] revealed a significant increase in LDH levels and the proportion of PI-positive cells in ZEA-treated gESCs, indicating that ZEA induced significant necrotic death of gESCs.

Accumulating evidence indicates that in addition to apoptosis and pyroptosis, necroptosis also occurs in various diseases, including stroke, neurodegenerative diseases, myocardial infarction, liver injury, and infectious disease [24]. RIPK1, RIPK3, and MLKL have been identified as key proteins that regulate necroptosis [25]. A recent study revealed that aflatoxin-B1 induced uterine injury in female mice and necrosis in the human endometrial microvasculature, accompanied by the upregulation of RIPK1, RIPK3, and MLKL [26]. However, whether necroptosis is involved in ZEA-induced necrotic death of gESCs has not been assessed. In our present study, we demonstrated that ZEA upregulated the mRNA expression of RIPK1 and RIPK3, as well as the phosphorylation of RIPK3 and MLKL in gESCs, indicating that ZEA treatments induced necroptosis in gESCs. Generally, phosphorylated RIPK1 and RIPK3 formed a necrosis signaling complex known as the necrosome, which further phosphorylates its MLKL substrate [27]. Activated MLKLs include autologous oligomers and translocate to the plasma membrane, resulting in necroptosis [28]. Moreover, studies have also provided evidence that mechanisms other than classical RIPK1/RIPK3/MLKL-mediated pathways regulate necroptosis, such as the RIPK3-, RIPK3-, and MLKL-, or MLKL-dependent pathways. Wen et al. reported that the RIPK3 inhibitor GSK’872 inhibited cell death. In contrast, the RIPK1 inhibitor Nec-1 did not affect zika-virus-infected astrocytes, indicating that zika-virus-induced necroptosis was RIPK3-dependent [29]. Zhang et al. reported that ceramide nanoliposomes targeted MLKL activation independently of the RIPK1/RIPK3-regulated pathway in ovarian cancer, and MLKL played a critical role in ceramide nanoliposome-induced necroptosis [30]. As expected, pretreatments with the RIPK1, RIPK3, and MLKL inhibitors Nec-1, GSK’872, and NSA, respectively, attenuated ZEA-induced gESCs LDH release. Moreover, NSA showed a dramatic inhibition efficiency, while Nec-1 and GSK’872 showed only a slight effect, indicating that gESCs’ necroptosis induced by ZEA depends mainly on the MLKL pathway. Moreover, the absence of changes in the mRNA level of MLKL, while the level of phosphorylated MLKL increased in the present study. These results suggested there may be other molecular mechanisms that activate MLKL in ZEA-induced necroptosis, such as post-translational modification, which require further investigation. Our data demonstrate that MLKL-mediated necroptosis is involved in ZEA-induced gESCs death.

Previous reports showed ROS production and mitochondrial damage were critical events in ZEA toxicity. Fu et al. found that ZEA promoted ROS overproduction in bovine mammary epithelial cells [31]. Furthermore, ROS induced by ZEA treatment was the critical factor in regulating cell cycle arrest and apoptosis in mouse Sertoli cells [15]. Moreover, Fan et al. demonstrated that ROS contributed to ZEA-induced mitochondrial damage in porcine IPEC-J2 cells [32]. According to these findings, we showed that ROS accumulates in ZEA-treated gESCs, and it is accompanied by a reduction in the mitochondrial membrane potential and increased ATP consumption. Pretreatments with the ROS scavenger NAC dramatically alleviated a ZEA-induced decrease in mitochondrial membrane potential and ATP content, indicating that ROS overproduction is the upstream regulator of mitochondrial dysfunction caused by ZEA in gESCs. Numerous studies have shown that ROS production and mitochondrial damage trigger or mediate necroptosis. Zhang et al. showed that mitochondrial ROS activate RIPK1 autophosphorylation, leading to a further recruitment of RIPK3 to form microsomes and resulting in necroptosis [33]. Sun et al. showed that ROS production and mitochondrial damage the downstream of the RIPK1-RIPK3 complex and trigger necroptosis in human colon cancer cells [34]. Moreover, Zhu et al. demonstrated that RIPK3 knockdown in cardiac ischemia-reperfusion (IR) injury inhibited necroptosis by blocking the Ca^2+^ overload-ROS-mPTT pathway [20]. In addition, ROS induced by iron overload interacted with the necrosome to regulate necroptosis in osteoblastic cells [35]. In the present study, we revealed that the MLKL inhibitor NSA markedly decreased ROS overproduction and attenuated the ZEA-induced decrease in mitochondrial membrane potential and ATP content in gESCs. These results suggest that mitochondrial dysfunction and ROS production are essential in ZEA-induced MLKL-driven necroptosis.

We also investigated how MLKL-mediated necroptosis affects ROS production in ZEA-treated gESCs. Ca^2+^ influx has been strongly linked with cell death caused by ZEA. Li et al. reported that ZEA promoted cytosolic Ca^2+^ accumulation in MLTC-1 cells, accompanied by mitochondrial dysfunction and ROS overproduction [36]. In addition, the chelation of cytosolic Ca^2+^ markedly decreased the endothelial cells’ apoptosis induced by ZEA [21]. In previous studies, our results confirmed that ZEA causes Ca^2+^ influx in a dose-dependent manner. Moreover, evidence suggests that phosphorylated MLKL forms a homotrimeric complex that translocates to the cytoplasmic membrane, forming a pore and regulating the ion channel [37,38]. Our results showed that consistent with the Ca^2+^ chelation, the inhibition of MLKL decreased the cytosolic Ca^2+^ overload, indicating that Ca^2+^ influxes were partly dependent on MLKL in ZEA-treated gESCs. In addition, studies implicated that Ca^2+^ influxes raised cellular ROS and mediated the mitochondrial damage, thus promoting cell necroptosis in cardiac IR injury, acute pancreatitis, and heart failure [20,39,40]. In the current study, we showed that cytosolic Ca^2+^ chelation with BAPTA-AM markedly inhibited the overproduction of ROS and alleviated the mitochondrial damage in gESCs caused by ZEA treatment. Taken together, our results prove that MLKL-mediated Ca^2+^ overload contributes to ROS generation and mitochondrial damage caused by ZEA treatments.

## 4. Materials and Methods

### 4.1. Materials

Dulbecco’s Modified Eagle Medium/F12 (DMEM/F12) was obtained from Hyclone (South Logan, UT, USA). Fetal bovine serum (FBS) was purchased from ZETA Life (Menlo Park, CA, USA). ZEA (Z2125) was obtained from Sigma-Aldrich (St. Louis, MO, USA). Nec-1 (HY-15760) and GSK’872 (HY-101872) were purchased from MedChem Express (Monmouth Junction, NJ, USA). NSA (T7129), NAC (T0875), BAPTA-AM (T6245), and cell counting kit-8 were purchased from TargetMol (Boston, MA, USA). Calcein/PI Cell Viability/Cytotoxicity assay kits, enhanced ATP assay kits, reactive oxygen species assay kits, and Fluo-4 AM were purchased from Beyotime Biotechnology (Beijing, China). JC-1 staining assay kits were purchased from Goyoo Biotechnology (Nanjing, China). Cytotoxicity LDH assay kits were obtained from Promega Biotechnology (Madison, WI, USA). The anti-P-RIPK3 (ser227, ab209384, 1:1000) antibody was purchased from Abcam (Cambridge, UK). The anti-P-MLKL (ser358, bsm-33331M, 1:1000) antibody was obtained from Bioss Biotechnology (Wuhan, China).

### 4.2. Cells Culture and Treatment

Immortalized goat endometrial stromal cells (gESCs), which were stored in our laboratory, were cultured in DMEM/F12 containing 10% FBS and 1% penicillin-streptomycin solution and incubated at 37 °C in a humidified atmosphere containing 5% CO_2_. ZEA was diluted in DMSO and added to the cell culture medium to indicate concentrations. To confirm the roles of necroptosis, ROS, and Ca^2+^ in ZEA-induced gESCs death, cells were separately pre-treated for 2 h with Nec-1, GSK’872, NSA, NAC, and BATPA-AM before co-treatment with ZEA for a further 24 h.

### 4.3. Cell Viability Assay

Cell viability was measured using the cell counting kit-8 method according to the manufacturer’s instructions. In brief, cells were seeded in 96-well plates at a density of 1 × 10^4^ cells/well. After treatment, 100 μL of fresh medium containing 10% CCK8 solution was added to each well, and the cells were incubated at 37 °C for 2 h. The absorbance of each well was measured at 450 nm using a microplate reader.

### 4.4. Cell Death Detection

To evaluate cell death, cytoplasmic enzyme lactate dehydrogenase (LDH) levels were measured using Cytotoxicity LDH assay kits. Briefly, culture supernatants were collected, and 50 μL was transferred to a fresh 96-well plate. After passing 50 μL of the CytoTox 96^®^ reagent to each well, the plate was incubated for 30 min on a plate shaker. Finally, after adding the 50 μL stop solution, the absorbance of each well was read at 490 nm using a microplate reader.

### 4.5. Calcein/PI Staining

Calcein-AM/PI staining was performed to assess cell necrosis according to the manufacturer’s protocol. In brief, cells were washed three times with PBS before incubation with calcein/PI reagents at 37 °C for 30 min in the absence of light. Images of the cells were captured under fluorescence microscopy.

### 4.6. RNA Isolation and Real-Time Quantitative PCR

To extract and synthesize cDNA from total RNA, RNAiso Plus (Takara Bio, Tokyo, Japan) and Evo M-MLV RT for PCR Kit (Accurate Biology, Hunan, China) were used, respectively. RT-qPCR was performed using the SYBE green plus reagent kit (BioRad, Hercules, CA, USA) according to the manufacturer’s instructions with the following primers: RIPK1, forward, 5′-CCAGCCTCAGAATCAACA-3′, reverse, 5′-AGAGCACAATGGCAAAGC-3′; RIPK3, forward, 5′-CGTAGAAGTGGCGGTCAA-3′, reverse, 5′-GGTAGCACATCCCGAGCA-3′; MLKL, forward, 5′-AGGCTGAGCGATGTCTGG-3′, reverse, 5′-CTGCTGGTCTTCCTGTTGC-3′; GAPDH, forward, 5′-GATGGTGAAGGTCGGAGTGAAC-3′, reverse, 5′-GTCATTGATGGCAACGATGT-3′. GAPDH was used as an invariant control, and the relative mRNA expression was calculated using the 2^−ΔΔCT^ method.

### 4.7. Western Blot Assay

After treatment, total proteins were extracted from cells using a cell lysis buffer for Western blot (Beyotime Biotechnology, Beijing, China), following the manufacturer’s instructions. The concentration of proteins was determined, and SDS-PAGE/immunoblotting was conducted as described previously [41]. The membrane was probed with appropriate antibodies, and protein bands were visualized using enhanced chemiluminescence (ECL) solution (Boster, Beijing, China). The intensity of protein bands was recorded digitally using Amersham ImageQuant 800 (Cytiva, Washington, DC, USA), and images were scanned and quantified using ImageJ software; β-actin was used as a loading control.

### 4.8. Mitochondrial Membrane Potential Assay

Changes in mitochondrial membrane potential were measured using the JC-1 staining assay kit according to the manufacturer’s instructions. Briefly, after the removal of the supernatant, the cells were washed twice with PBS and incubated with JC-1 reagent at 37 °C for 25 min. After two washes with staining buffer, fluorescent images of the cells were captured using a fluorescence microscope or a fluorescence microplate (Axio Observer, Carl Zeiss AG, Oberkochen, Germany).

### 4.9. Reactive Oxygen Species (ROS) Assay

Cellular ROS production was measured with an ROS assay kit according to the manufacturer’s instructions. After treatment, the cells were incubated with DCFH-DA diluted in a medium without serum (1:1000) for 20 min at 37 °C. Following three washes with a medium, ROS production was observed and recorded under a fluorescence microscope and a fluorescence microplate.

### 4.10. ATP Assay

As directed by the manufacturer, ATP contents were determined using an enhanced ATP assay kit. After treatment with the ATP lysis solution, the lysate was centrifuged at 12,000× *g* for 5 min at 4 °C, and the supernatant was collected. An ATP-detection working solution (100 μL) was added to each well and incubated for 5 min of incubation at room temperature before adding 50 μL of the supernatant or standard samples to each well. The luminescence signal (RLU) was measured with a Luminometer.

### 4.11. Statistical Analysis

All data were expressed as mean ± standard deviation (SD). One-way analysis of variance (ANOVA) was used to determine the statistical significance of differences between groups of GraphPad Prism TM 7 (GraphPad Software Inc., La Jolla, CA, USA). All experiments were performed at least three times.

## 5. Conclusions

Our results demonstrated that ZEA could induce necroptosis in gESCs. Mechanistically, ZEA promoted MLKL phosphorylation, leading to Ca^2+^ influx, ROS accumulation, and mitochondrial dysfunction, which caused necroptosis (Figure 5G). Thus, this study extends our understanding of ZEA cytotoxicity’s underlying molecular mechanisms and provides a valuable therapeutic strategy for ZEA poisoning.

## Data Availability

Not applicable.
